# Supplementary material for: PTEN‐mediated dephosphorylation of 53BP1 confers cellular resistance to DNA damage in cancer cells
Source: Mol Oncol. 2023 Dec 12;18(3):580–605. doi: 10.1002/1878-0261.13563 (PMC10920079; doi:10.1002/1878-0261.13563)
Supplement: Supplementary file 1 — Fig. S1. PTEN promotes HR repair through enhancing DNA end resection. [file MOL2-18-580-s005.pdf]

He_Fig. S1

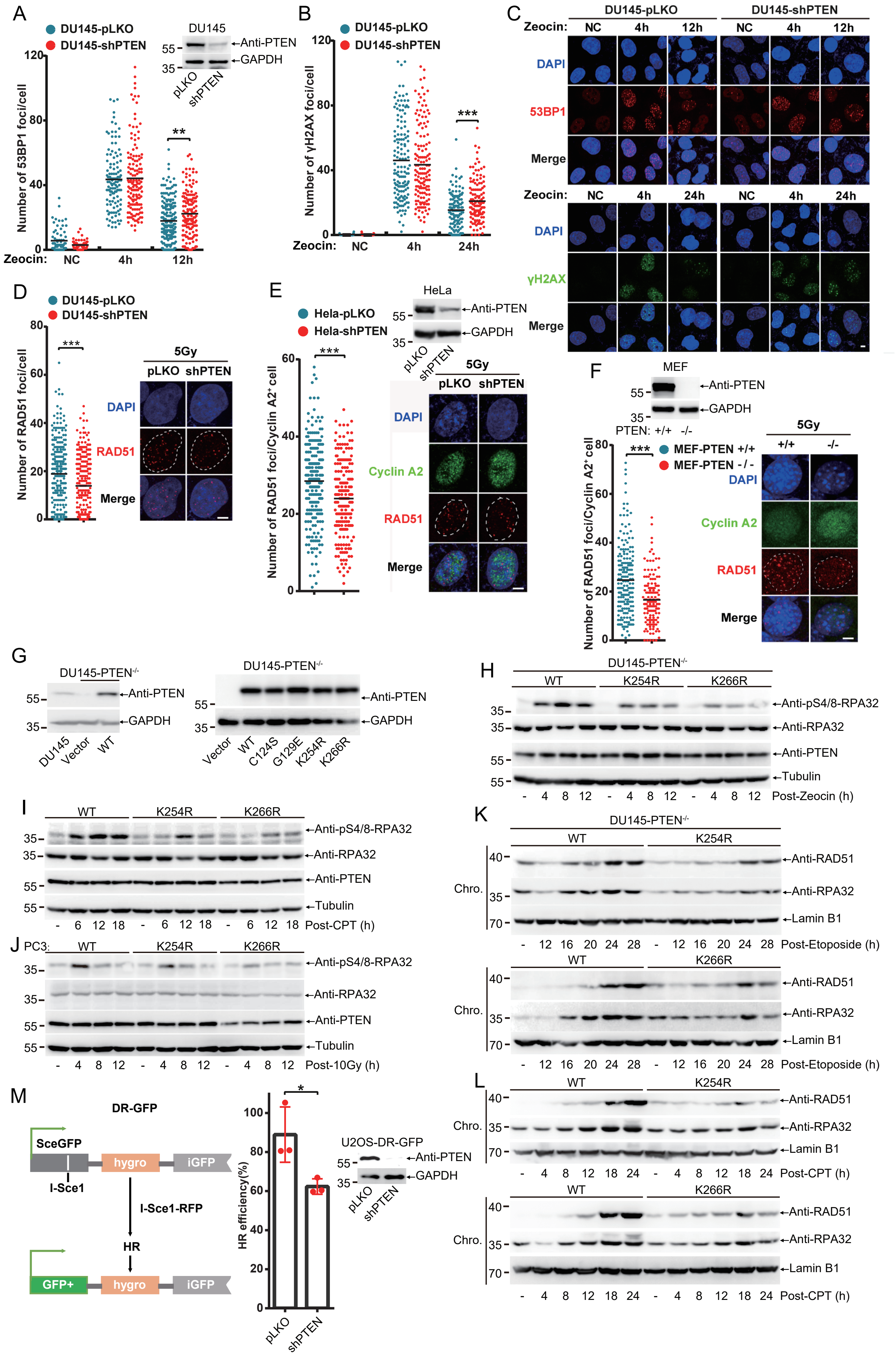
A
DU145-pLKO
DU145-shPTEN
DU145
Anti-PTEN
GAPDH
pLKO
shPTEN
Number of 53BP1 foci/cell
Zeocin: NC 4h 12h
B
DU145-pLKO
DU145-shPTEN
Number of γH2AX foci/cell
Zeocin: NC 4h 24h
C
DU145-pLKO
DU145-shPTEN
Zeocin: NC 4h 12h NC 4h 12h
DAPI
53BP1
Merge
Zeocin: NC 4h 24h NC 4h 24h
DAPI
γH2AX
Merge
D
DU145-pLKO
DU145-shPTEN
Number of RAD51 foci/cell
5Gy
pLKO shPTEN
DAPI
RAD51
Merge
E
Hela-pLKO
Hela-shPTEN
HeLa
Anti-PTEN
GAPDH
pLKO
shPTEN
Number of RAD51 foci/Cyclin A2+ cell
5Gy
pLKO shPTEN
DAPI
Cyclin A2
RAD51
Merge
F
MEF
Anti-PTEN
GAPDH
PTEN: +/+ -/-
MEF-PTEN +/+
MEF-PTEN -/-
Number of RAD51 foci/Cyclin A2+ cell
5Gy
+/+ -/-
DAPI
Cyclin A2
RAD51
Merge
G
DU145-PTEN-/-
Anti-PTEN
GAPDH
DU145 Vector WT
DU145-PTEN-/-
Anti-PTEN
GAPDH
Vector WT C124S G129E K254R K266R
H
DU145-PTEN-/-
WT K254R K266R
Anti-pS4/8-RPA32
Anti-RPA32
Anti-PTEN
Tubulin
- 4 8 12 - 4 8 12 - 4 8 12 Post-Zeocin (h)
I
WT K254R K266R
Anti-pS4/8-RPA32
Anti-RPA32
Anti-PTEN
Tubulin
- 6 12 18 - 6 12 18 - 6 12 18 Post-CPT (h)
J
PC3: WT K254R K266R
Anti-pS4/8-RPA32
Anti-RPA32
Anti-PTEN
Tubulin
- 4 8 12 - 4 8 12 - 4 8 12 Post-10Gy (h)
K
DU145-PTEN-/-
WT K254R
Chro.
Anti-RAD51
Anti-RPA32
Lamin B1
- 12 16 20 24 28 - 12 16 20 24 28 Post-Etoposide (h)
WT K266R
Chro.
Anti-RAD51
Anti-RPA32
Lamin B1
- 12 16 20 24 28 - 12 16 20 24 28 Post-Etoposide (h)
L
WT K254R
Chro.
Anti-RAD51
Anti-RPA32
Lamin B1
- 4 8 12 18 24 - 4 8 12 18 24 Post-CPT (h)
WT K266R
Chro.
Anti-RAD51
Anti-RPA32
Lamin B1
- 4 8 12 18 24 - 4 8 12 18 24 Post-CPT (h)
M
DR-GFP
SceGFP
hygro
iGFP
I-Sce1
I-Sce1-RFP
HR
GFP+
hygro
iGFP
HR efficiency(%)
pLKO
shPTEN
U2OS-DR-GFP
Anti-PTEN
GAPDH
pLKO
shPTEN

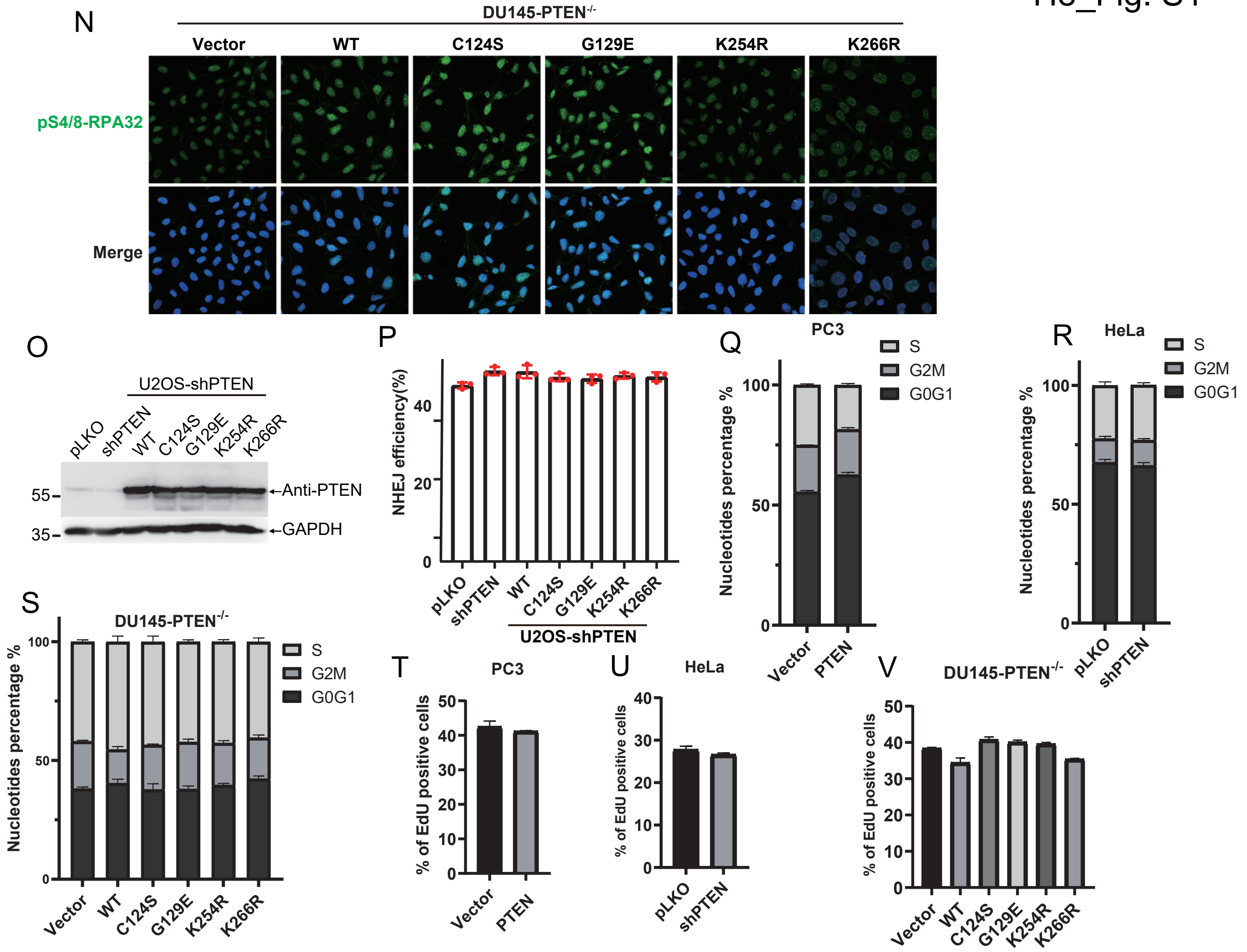

Fig. S1 PTEN promotes HR repair through enhancing DNA end resection. (A-C) Quantification of 53BP1 and γH2AX foci in DU145 cells after treatment with Zeocin (200 μg/mL) for 1 h and recovery for indicated time (53BP1: DU145-pLKO(n(NC)=74, n(4h)=121, n(12h)=205), DU145-shPTEN(n(NC)=66, n(4h)=143, n(12h)=154); γH2AX: DU145-pLKO(n(NC)=36, n(4h)=142, n(24h)=134), DU145-shPTEN(n(NC)=63, n(4h)=141, n(24h)=130)). Inset: immunoblot of PTEN knockdown efficiency in DU145 cells. Representative immunofluores-cence images were shown. scale bar, 20 μm. (D-F) Quantification of RAD51 foci in DU145, HeLa and MEF cells after treatment with 5 Gy and recovery for 6 h (DU145-pLKO(n=259), DU145-shPTEN(n=266); HeLa-pLKO(n=235), HeLa-shPTEN(n=203); MEF-PTEN$^{+/+}$(n=196), MEF-PTEN$^{-/-}$(n=144)). Cyclin A2 was used to indicate S or G2 phase cells. Representative immunofluorescence images were shown. Inset: immunoblot of PTEN knockdown or knockout efficiency in HeLa and MEF cells. scale bar, 20 μm. (G) Immunoblot of PTEN in DU145, DU145-PTEN$^{-/-}$ cells and stably re-expressing PTEN-WT, C124S, G129E, K254R and K266R. (H-I) Immunoblot of pS4/8-RPA32 level in DU145-PTEN$^{-/-}$ cells stably re-expressing PTEN-WT, K254R and K266R after treatment with CPT (20 μM) or Zeocin (200 μg/mL) for 1h and recovery for indicated time. (J) Immunoblot of pS4/8-RPA32 level in PC3 cells stably re-expressing PTEN-WT, K254R and K266R after treatment with 10 Gy and recovery for indicated time. (K, L) Immunoblot of chromatin associated RAD51 and RPA32 proteins separated from DU145 cells after treatment with Etoposide (30 μM) or CPT (20 μM) for 1 h and recovery for indicated time. (M) Left panel describes how DR-GFP reporter works. HR efficiency were quantified in U2OS-DR-GFP and U2OS-DR-GFP-shPTEN cells after transfection of I-Sce1 for 48-72h at right panel (n=3 for each group). Inset: immunoblot of PTEN knockdown efficiency in U2OS-DR-GFP cells. Unpaired Student's t-test was used (*$p< 0.05$,**$p< 0.01$, ***$p< 0.001$) and data were shown as mean or mean±s.d. (N) Immunofluorescence of p-RPA32 was detected in DU145-PTEN$^{-/-}$ cells and stably re-expressing PTEN-WT, K254R and K266R cells at 4 h after 5Gy. (O) Expression level of PTEN in U2OS-pLKO, U2OS-shPTEN and stably re-expressing PTEN-WT, C124S, G129E, K254R and K266R cells. (P) NHEJ efficiency were quantified in U2OS-pLKO, U2OS-shPTEN and stably re-expressing PTEN-WT, C124S, G129E, K254R and K266R cells after transfection of EJ5-GFP and I-Sce1 for 48h (n=3 for each group). (Q-S) Cell cycle profile was detected in PC3, HeLa, and DU145 cells. (T-V) Edu incorporation efficiency was detected in PC3, HeLa, and DU145 cells.
