# Supplementary material for: PTEN‐mediated dephosphorylation of 53BP1 confers cellular resistance to DNA damage in cancer cells
Source: Mol Oncol. 2023 Dec 12;18(3):580–605. doi: 10.1002/1878-0261.13563 (PMC10920079; doi:10.1002/1878-0261.13563)
Supplement: Supplementary file 2 — Fig. S2. DNA damage promotes PTEN chromatin loading by inducing its SUMOylation. [file MOL2-18-580-s004.pdf]

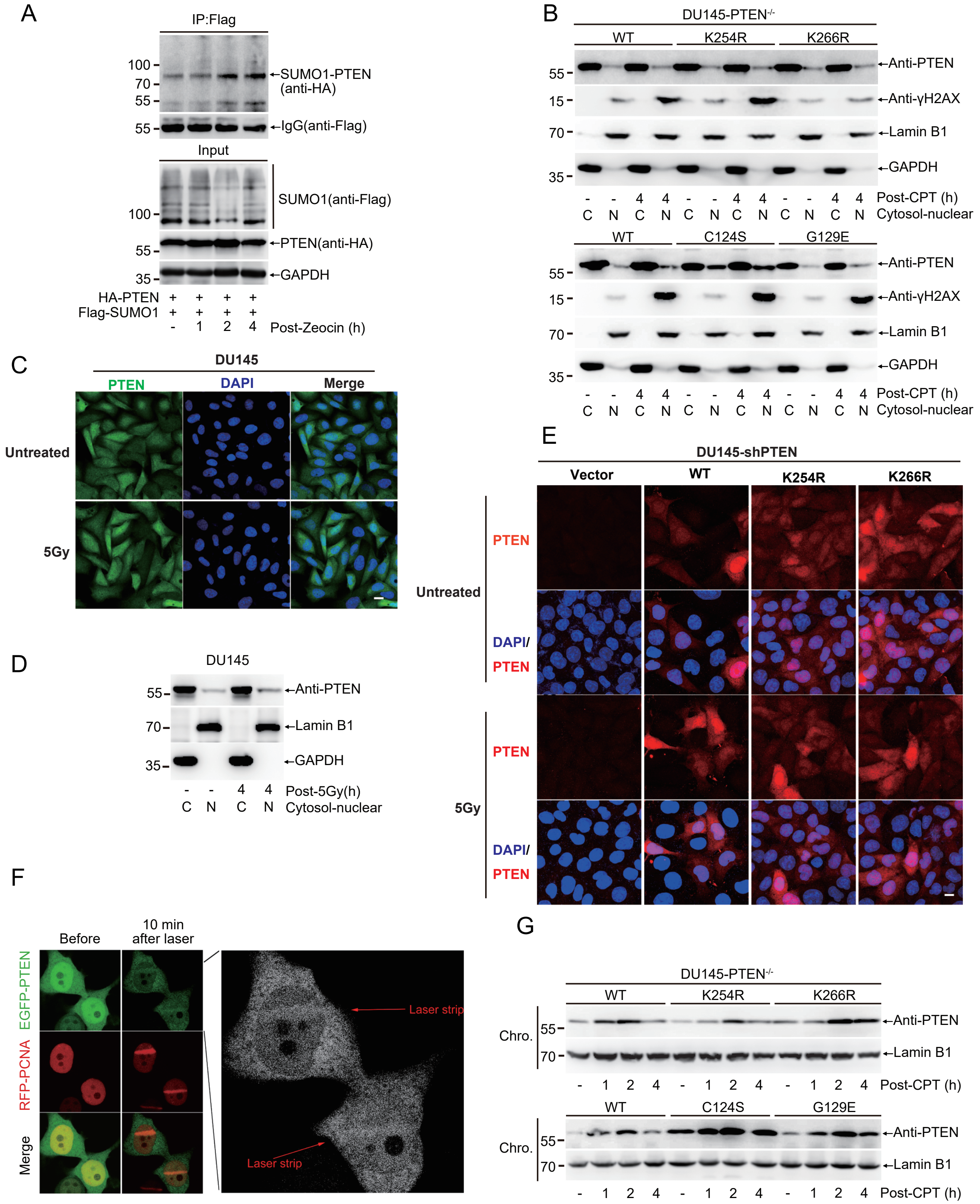
A
IP:Flag
100
70
55
SUMO1-PTEN (anti-HA)
55
IgG(anti-Flag)
Input
100
SUMO1(anti-Flag)
55
PTEN(anti-HA)
35
GAPDH
HA-PTEN + + + +
Flag-SUMO1 + + + +
- 1 2 4 Post-Zeocin (h)
B
DU145-PTEN-/-
WT K254R K266R
55 Anti-PTEN
15 Anti-γH2AX
70 Lamin B1
35 GAPDH
- - 4 4 - - 4 4 - - 4 4 Post-CPT (h)
C N C N C N C N C N C N Cytosol-nuclear
WT C124S G129E
55 Anti-PTEN
15 Anti-γH2AX
70 Lamin B1
35 GAPDH
- - 4 4 - - 4 4 - - 4 4 Post-CPT (h)
C N C N C N C N C N C N Cytosol-nuclear
C
DU145
PTEN DAPI Merge
Untreated
5Gy
D
DU145
55 Anti-PTEN
70 Lamin B1
35 GAPDH
- - 4 4 Post-5Gy(h)
C N C N Cytosol-nuclear
E
DU145-shPTEN
Vector WT K254R K266R
Untreated
PTEN
DAPI/ PTEN
5Gy
PTEN
DAPI/ PTEN
F
Before
10 min after laser
EGFP-PTEN
RFP-PCNA
Merge
Laser strip
Laser strip
G
DU145-PTEN-/-
WT K254R K266R
Chro.
55 Anti-PTEN
70 Lamin B1
- 1 2 4 - 1 2 4 - 1 2 4 Post-CPT (h)
WT C124S G129E
Chro.
55 Anti-PTEN
70 Lamin B1
- 1 2 4 - 1 2 4 - 1 2 4 Post-CPT (h)

Fig. S2 DNA damage promotes PTEN chromatin loading by inducing its SUMOylation. (A) Denatured Co-IP were performed to analyze SUMOylation of PTEN in 293T cells transfected with Flag-SUMO1 and HA-PTEN after treatment with Zeocin (400 μg/mL). (B) PTEN localization was detected with immunoblot after nuclear-cytosol separation in DU145-PTEN$^{-/-}$ cells stably re-expressing PTEN mutants after treatment with CPT (20 μM) for 1 h and recovery for 4 h. (C) Immunoflurescence of PTEN were detected in DU145 cells with or without treatment with 5Gy at 4 h. (D) Nuclear-cytosol fractionation was performed in DU145 cells at 4 h with or without treatment with 5Gy and PTEN was detedcted. (E) Immunoflurescence of PTEN were detected in DU145-PTEN$^{-/-}$ cells reconstituted with PTEN-WT, K254R and K266R at 4 h with or without treatment with 5Gy. (F) 293T$^{senp-/-}$ cells transfected with PCNA-RFP and EGFP-PTEN were treated with laser micro-irradiation. Images were taken before and 10 min post laser micro-irradiation. PCNA-RFP were used as indicator of DNA damage location. (G) Chromatin loading of PTEN was detedcted with immunoblot in DU145-PTEN$^{-/-}$ cells stably re-expressing PTEN mutants after treatment with CPT (20 μM) for 1 h and recovery for indicated time.
