# Supplementary material for: PTEN‐mediated dephosphorylation of 53BP1 confers cellular resistance to DNA damage in cancer cells
Source: Mol Oncol. 2023 Dec 12;18(3):580–605. doi: 10.1002/1878-0261.13563 (PMC10920079; doi:10.1002/1878-0261.13563)
Supplement: Supplementary file 3 — Fig. S3. p14ARF is a novel SUMO E3 ligase to mediate PTEN SUMOylation during DDR. [file MOL2-18-580-s002.pdf]

He_Fig. S3

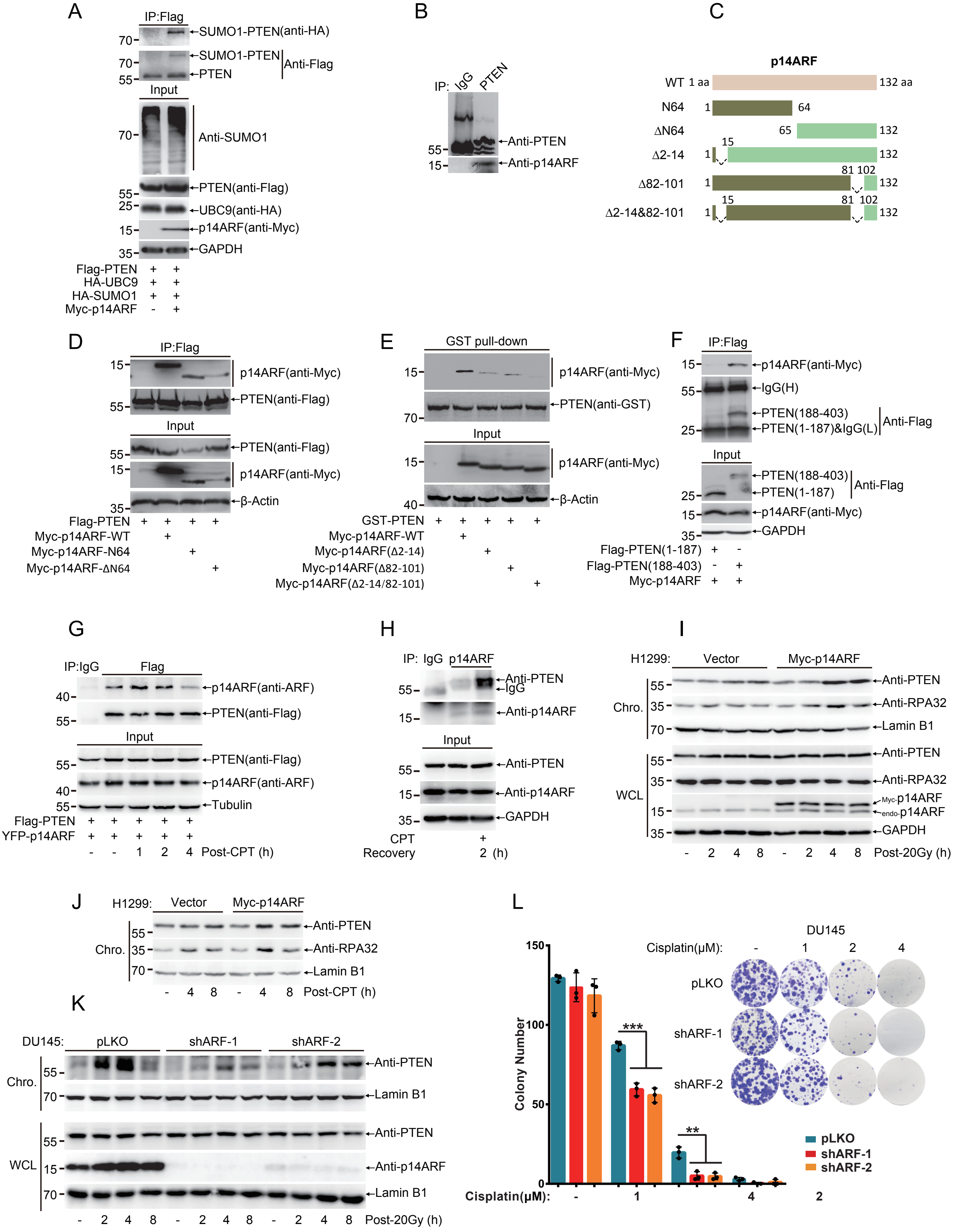

A
IP:Flag
70
SUMO1-PTEN(anti-HA)
70
55
SUMO1-PTEN
PTEN
Anti-Flag
Input
70
Anti-SUMO1
55
PTEN(anti-Flag)
25
UBC9(anti-HA)
15
p14ARF(anti-Myc)
35
GAPDH
Flag-PTEN + +
HA-UBC9 + +
HA-SUMO1 + +
Myc-p14ARF - +
B
IP: IgG PTEN
55
Anti-PTEN
15
Anti-p14ARF
C
p14ARF
WT 1 aa 132 aa
N64 1 64
ΔN64 65 132
Δ2-14 1 15 132
Δ82-101 1 81 102 132
Δ2-14&82-101 1 15 81 102 132
D
IP:Flag
15
p14ARF(anti-Myc)
55
PTEN(anti-Flag)
Input
55
PTEN(anti-Flag)
15
p14ARF(anti-Myc)
35
β-Actin
Flag-PTEN + + + +
Myc-p14ARF-WT +
Myc-p14ARF-N64 +
Myc-p14ARF-ΔN64 +
E
GST pull-down
15
p14ARF(anti-Myc)
70
PTEN(anti-GST)
Input
15
p14ARF(anti-Myc)
40
β-Actin
GST-PTEN + + + + +
Myc-p14ARF-WT +
Myc-p14ARF(Δ2-14) +
Myc-p14ARF(Δ82-101) +
Myc-p14ARF(Δ2-14/82-101) +
F
IP:Flag
15
p14ARF(anti-Myc)
55
IgG(H)
25
PTEN(188-403)
PTEN(1-187)&IgG(L)
Anti-Flag
Input
25
PTEN(188-403)
PTEN(1-187)
Anti-Flag
15
p14ARF(anti-Myc)
35
GAPDH
Flag-PTEN(1-187) + -
Flag-PTEN(188-403) - +
Myc-p14ARF + +
G
IP:IgG Flag
40
p14ARF(anti-ARF)
55
PTEN(anti-Flag)
Input
55
PTEN(anti-Flag)
40
p14ARF(anti-ARF)
55
Tubulin
Flag-PTEN + + + + +
YFP-p14ARF + + + + +
- - 1 2 4 Post-CPT (h)
H
IP: IgG p14ARF
55
Anti-PTEN
IgG
15
Anti-p14ARF
Input
55
Anti-PTEN
15
Anti-p14ARF
35
GAPDH
CPT +
Recovery 2 (h)
I
H1299: Vector Myc-p14ARF
Chro.
55
Anti-PTEN
35
Anti-RPA32
70
Lamin B1
WCL
55
Anti-PTEN
35
Anti-RPA32
15
Myc-p14ARF
endo-p14ARF
35
GAPDH
- 2 4 8 - 2 4 8 Post-20Gy (h)
J
H1299: Vector Myc-p14ARF
Chro.
55
Anti-PTEN
35
Anti-RPA32
70
Lamin B1
- 4 8 - 4 8 Post-CPT (h)
K
DU145: pLKO shARF-1 shARF-2
Chro.
55
Anti-PTEN
70
Lamin B1
WCL
55
Anti-PTEN
15
Anti-p14ARF
70
Lamin B1
- 2 4 8 - 2 4 8 - 2 4 8 Post-20Gy (h)
L
150
100
50
0
Colony Number
***
**
Cisplatin(μM): - 1 4 2
DU145
Cisplatin(μM): - 1 2 4
pLKO
shARF-1
shARF-2
pLKO
shARF-1
shARF-2

Fig. S3 p14ARF is a novel SUMO E3 ligase to mediate PTEN SUMOylation during DDR. (A) SUMOylated PTEN was detected with IP in 293T cells overexpressed indicated plasmids. (B) Reciprocal endogenous interaction between PTEN and p14ARF were detected with Co-IP in 293T cells. (C) Schematic structure of truncated p14ARF. (D) Truncated Myc-p14ARF and Flag-PTEN were transfected into 293T cells for 48 h, interaction domain between PTEN and p14ARF were identified with Co-IP. (E) GST-PTEN was purified from BL21 and incubated with lysis of 293T cells transfected with different truncated Myc-p14ARF, PTEN bound truncated Myc-p14ARF was identified with GST pull-down and immunoblot. (F) Truncated Flag-PTEN and Myc-p14ARF were transfected into 293T cells for 48 h, domain of PTEN responsible for interacting with p14ARF were identified with Co-IP. (G) Interaction between PTEN and p14ARF after treatment with CPT (20 μM) for 1 h and recovery for indicated time was identified with Co-IP. (H) Co-IP was performed to detected endogenous interaction in Du145 cell after CPT treatment. (I, J) Chromatin loaded PTEN were separated from H1299 stably expressing Vector or Myc-p14ARF after treatment with 20 Gy or CPT (20 μM) for 1 h and recovery for indicated time. (K) Immunoblot of chromatin loaded PTEN which separated from DU145-pLKO, shARF-1 and shARF-2 cells after treatment with 20 Gy and recovery for indicated time. (L) Colony survival of DU145-pLKO, shARF-1 and shARF-2 cells after treatment with different doses of Cisplatin. Colony number was counted and shown as mean±s.d. from three independent experiments at left panel. Representative colony images were shown at right panel. Unpaired Student's t-test was used (**$p < 0.01$, ***$p < 0.001$).
