# Supplementary material for: PTEN‐mediated dephosphorylation of 53BP1 confers cellular resistance to DNA damage in cancer cells
Source: Mol Oncol. 2023 Dec 12;18(3):580–605. doi: 10.1002/1878-0261.13563 (PMC10920079; doi:10.1002/1878-0261.13563)
Supplement: Supplementary file 4 — Fig. S4. PTEN relieves HR barrier posted by 53BP1 through directly dephosphorylating pT543‐53BP1. [file MOL2-18-580-s007.pdf]

He_Fig. S4

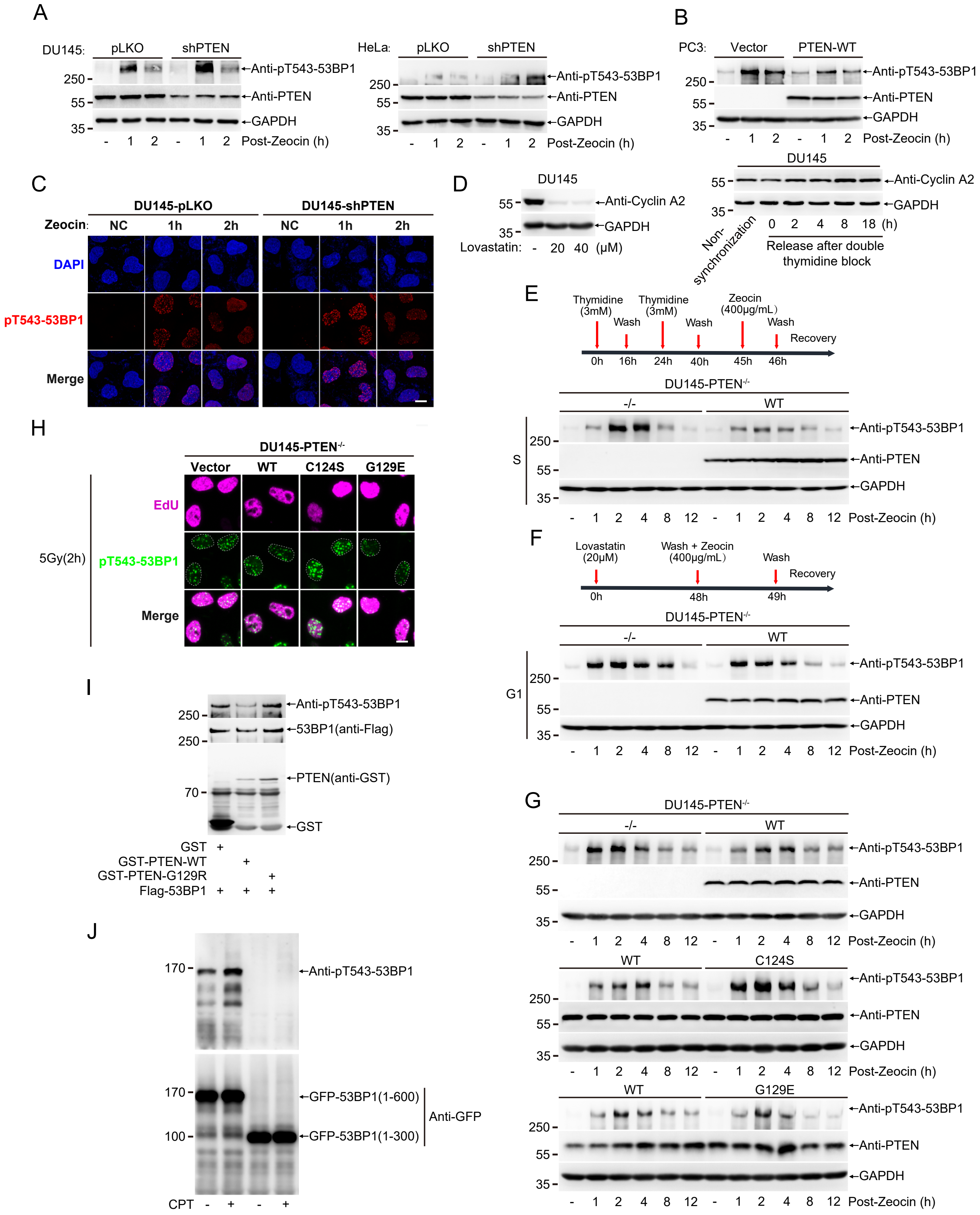
A
DU145:
pLKO
shPTEN
Anti-pT543-53BP1
Anti-PTEN
GAPDH
250
55
35
- 1 2 - 1 2 Post-Zeocin (h)
HeLa:
pLKO
shPTEN
Anti-pT543-53BP1
Anti-PTEN
GAPDH
- 1 2 - 1 2 Post-Zeocin (h)
B
PC3:
Vector
PTEN-WT
Anti-pT543-53BP1
Anti-PTEN
GAPDH
- 1 2 - 1 2 Post-Zeocin (h)
DU145
Anti-Cyclin A2
GAPDH
Non-synchronization
0 2 4 8 18 (h)
Release after double thymidine block
C
DU145-pLKO
DU145-shPTEN
Zeocin:
NC 1h 2h NC 1h 2h
DAPI
pT543-53BP1
Merge
D
DU145
Anti-Cyclin A2
GAPDH
Lovastatin: - 20 40 (μM)
E
Thymidine (3mM)
Wash
Thymidine (3mM)
Wash
Zeocin (400μg/mL)
Wash
Recovery
0h 16h 24h 40h 45h 46h
DU145-PTEN-/-
-/-
WT
S
Anti-pT543-53BP1
Anti-PTEN
GAPDH
- 1 2 4 8 12 - 1 2 4 8 12 Post-Zeocin (h)
F
Lovastatin (20μM)
Wash + Zeocin (400μg/mL)
Wash
Recovery
0h 48h 49h
DU145-PTEN-/-
-/-
WT
G1
Anti-pT543-53BP1
Anti-PTEN
GAPDH
- 1 2 4 8 12 - 1 2 4 8 12 Post-Zeocin (h)
G
DU145-PTEN-/-
-/-
WT
Anti-pT543-53BP1
Anti-PTEN
GAPDH
- 1 2 4 8 12 - 1 2 4 8 12 Post-Zeocin (h)
WT
C124S
Anti-pT543-53BP1
Anti-PTEN
GAPDH
- 1 2 4 8 12 - 1 2 4 8 12 Post-Zeocin (h)
WT
G129E
Anti-pT543-53BP1
Anti-PTEN
GAPDH
- 1 2 4 8 12 - 1 2 4 8 12 Post-Zeocin (h)
H
DU145-PTEN-/-
Vector WT C124S G129E
5Gy(2h)
EdU
pT543-53BP1
Merge
I
Anti-pT543-53BP1
53BP1(anti-Flag)
PTEN(anti-GST)
GST
250
250
70
GST +
GST-PTEN-WT +
GST-PTEN-G129R +
Flag-53BP1 + + +
J
170
170
100
Anti-pT543-53BP1
GFP-53BP1(1-600)
GFP-53BP1(1-300)
Anti-GFP
CPT - + - +

Fig. S4 PTEN relieves HR barrier posted by 53BP1 through directly dephosphorylating pT543-53BP1. (A) Immunoblot of pT543-53BP1 level in DU145 and HeLa cells stably expressing pLKO or shPTEN after treatment with Zoecin (200 μg/mL) for 1 h and recovery for indicated time. (B) Immunoblot of pT543-53BP1 level in PC3 cells stably expressing Vector or PTEN-WT after treatment with Zoecin (200 μg/mL) for 1 h and recovery for indicated time. (C) Representative images corresponding to Fig. 4B. (D) Cell cycle were synchronized to G1 with lovastatin or to S phase with double thymidine block in DU145 cells. Immunoblot of Cyclin A2 was shown to detect the synchronization efficiency. (E) Schematic illustration of S phase synchronization and Zeocin addition was shown at upper panel. Immunoblot of pT543-53BP1 level in DU145 cells after S synchronization was shown at lower panel. (F) Schematic illustration of G1 phase synchronization and Zeocin addition was shown at upper panel. Immunoblot of pT543-53BP1 level in DU145 cells after G1 synchronization was shown at lower panel. (G) Immunoblot of pT543-53BP1 level in DU145 cells after Zeocin (400 μg/mL) treatment for 1 h and recovery for indicated time. (H) Representative images corresponding to Fig. 4H. (I) Immunoblot of total pT543-53BP1 level after in vitro dephosphorylation assay. Full-length Flag-53BP1 were immunoprecipitated from 293T cells after treatment with CPT (20 μM) and recovery for 1 h and then reacted with purified GST-PTEN-WT and PTEN-G129R (a dual phosphatase deficient mutant) which were purified from BL21. (J) Immunoblot of pT543-53BP1 from 293T cells which were transfected GFP-53BP1(1-300) or GFP-53BP1(1-600) after treatment with CPT (20 μM) for 1 h and recovery for 1 h.
