# Supplementary material for: PTEN‐mediated dephosphorylation of 53BP1 confers cellular resistance to DNA damage in cancer cells
Source: Mol Oncol. 2023 Dec 12;18(3):580–605. doi: 10.1002/1878-0261.13563 (PMC10920079; doi:10.1002/1878-0261.13563)
Supplement: Supplementary file 5 — Fig. S5. PTEN chromatin loading is mediated by BRCA1 recruiting SUMOylated PTEN via its N‐terminal SIM. [file MOL2-18-580-s001.pdf]

He_Fig. S5

A

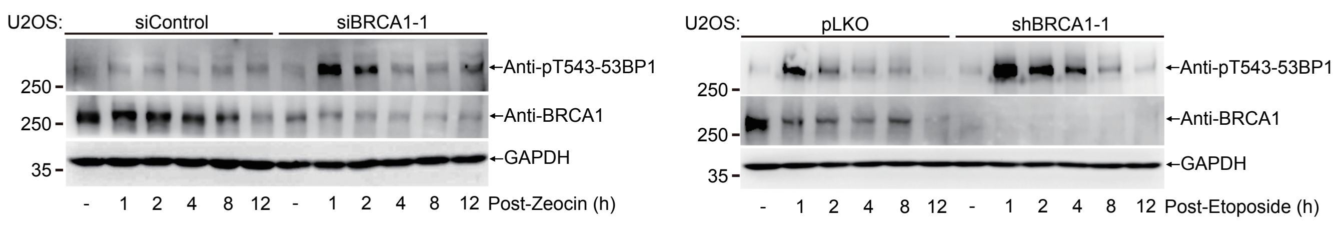

B

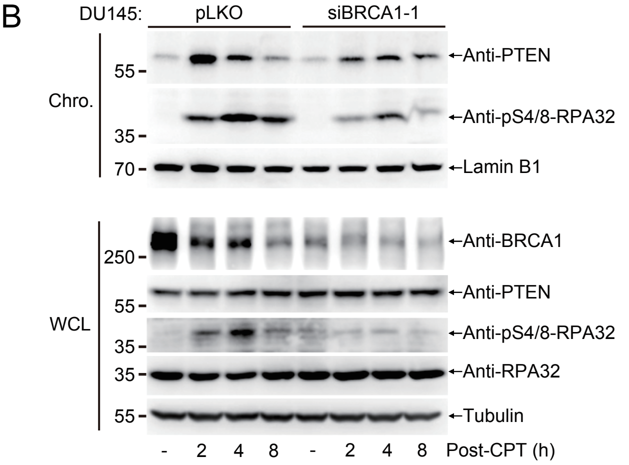

C

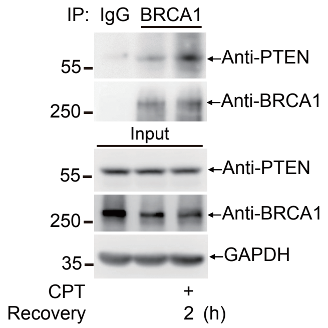

D

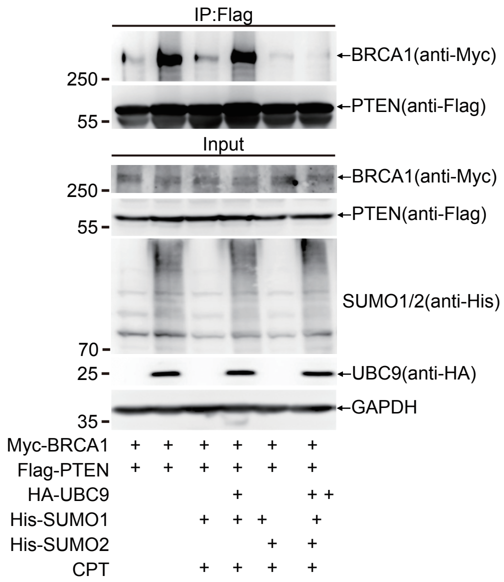

E JASSA

Exon11 (AA 204-207 to AA 1086-1089)

Position site	Sequence	Type	α/S stretch	PS	DB Hit
AA 28-31	KILECPICLELIKEPVSTKC	SIM Type 2	[N][SIM][N]	0.022	
AA 49-52	HIFCKFCMLKLLNQKKGPSQ	SIM Type 2	[N][SIM][N]	0	
AA 86-89	RFSQLVEELLKIICAFQLDT	SIM Type 1	[N][SIM][N]	0	
AA 87-90	FSQLVEELLKIICAFQLDTG	SIM Type 2	[N][SIM][N]	0	
AA 122-125	SPEHLKDEVSIIQSMGYRNR	SIM Type α	[Y][SIM][N]	0.868	1
AA 204-207	YCSVGDQELLQITPQGTRDE	SIM Type 1	[N][SIM][N]	0	
AA 289-292	SSLQHENSSLLLTKDRMNVE	SIM Type 4	[Y][SIM][N]	0	
AA 412-415	ESNAKVADVLDVLNEVDEYS	SIM Type 1	[Y][SIM][Y]	2.585	
AA 413-416	SNAKVADVLDVLNEVDEYSG	SIM Type 2	[N][SIM][Y]	0.137	
AA 429-432	EYSGSSEKIDLLASDPHEAL	SIM Type 2	[Y][SIM][N]	0.107	1
AA 480-483	PNLSHVTENLIIGAFVTEPQ	SIM Type 4	[N][SIM][N]	0	
AA 623-626	SSTRHIHALELVVSRNLSPP	SIM Type 2	[N][SIM][N]	0.021	
AA 624-627	STRHIHALELVVSRNLSPPN	SIM Type 4	[N][SIM][N]	0.103	
AA 766-772	ERSVESSSISLVPGTDYGTQ	SIM Type r	[Y][SIM][N]	0.094	
AA 783-786	TDYGTQESISLLEVSTLGKA	SIM Type 2	[Y][SIM][N]	0.161	
AA 785-788	YGTQESISLLEVSTLGKAKT	SIM Type 1	[Y][SIM][N]	0.094	1
AA 1086-1089	PKLNAMLRLGVLQPEVYKQS	SIM Type 2	[N][SIM][N]	0.001	
AA 1260-1263	LSKNTEENLLSLKNSLNDCS	SIM Type 1	[N][SIM][N]	0.018	
AA 1273-1276	NSLNDCSNQVILAKASQEHH	SIM Type 4	[N][SIM][N]	0.002	
AA 1404-1407	QRDTMQHNLIKLQQEMAELE	SIM Type 1	[N][SIM][N]	0.002	
AA 1528-1531	RNYPSQEELIKVVDVEEQQL	SIM Type 1	[Y][SIM][Y]	0.001	
AA 1529-1532	NYPSQEELIKVVDVEEQQLE	SIM Type α	[Y][SIM][Y]	0.001	
AA 1531-1534	PSQEELIKVVDVEEQQLEES	SIM Type β	[Y][SIM][Y]	10.774	
AA 1806-1809	FTLGTGVHPIVVVQPDAWTE	SIM Type 4	[N][SIM][N]	1.789	
AA 1807-1810	TLGTGVHPIVVVQPDAWTED	SIM Type α	[N][SIM][N]	3.012	

F GPS-SUMO

Exon11 (204 - 208 to 1116 - 1120)

Position	Peptide	Score	Cutoff	P-value	Type
11-15	SALRVEE VQNVI NAMQKIL	2.161	0	0.943	SUMO Interaction
28 - 32	ILECPIC LELIK EPVSTKC	13.882	0	0.6	SUMO Interaction
49 - 53	IFCKFCM LKLLN QKKGPSQ	3.36	0	0.956	SUMO Interaction
87 - 91	SQLVEEL LKIIC AFQLDTG	14.753	0	0.645	SUMO Interaction
122 - 126	PEHLKDE VSIIQ SMGYRNR	29.725	0	0.216	SUMO Interaction
152 - 156	PSLQETS LSVQL SNLGTVR	5.205	0	0.901	SUMO Interaction
178 - 182	IQPQKTS VYIEL GSDSSED	14.246	0	0.662	SUMO Interaction
204 - 208	CSVGDQE LLQIT PQGTRDE	27.335	0	0.199	SUMO Interaction
290 - 294	LQHENSS LLLTK DRMNVEK	5.084	0	0.919	SUMO Interaction
412 - 416	SNAKVAD VLDVL NEVDEYS	24.954	0	0.412	SUMO Interaction
429 - 433	YSGSSEK IDLLA SDPHEAL	28.305	0	0.217	SUMO Interaction
481 - 485	LSHVTEN LIIGA FVTEPQI	19.235	0	0.41	SUMO Interaction
596 - 600	SSISNME LELNI HNSKAPK	6.954	0	0.873	SUMO Interaction
623 - 627	STRHIHA LELVV SRNLSPP	21.746	0	0.368	SUMO Interaction
769 - 773	RSVESSS ISLVP GTDYGTQ	15.938	0	0.481	SUMO Interaction
783 - 787	DYGTQES ISLLE VSTLGKA	22.034	0	0.364	SUMO Interaction
1086 - 1090	KLNAMLR LGVLQ PEVYKQS	15.277	0	0.413	SUMO Interaction
1116 - 1120	KKQEYEE VVQTV NTDFSPY	6.238	0	0.807	SUMO Interaction
1260 - 1264	SKNTEEN LLSLK NSLNDCS	20.739	0	0.436	SUMO Interaction
1274 - 1278	LNDCSNQ VILAK ASQEHHL	16.588	0	0.465	SUMO Interaction
1404 - 1408	RDTMQHN LIKLQ QEMAELE	20.66	0	0.362	SUMO Interaction
1414 - 1418	LQQEMAE LEAVL EQHGSQP	12.344	0	0.567	SUMO Interaction
1529 - 1533	YPSQEEL IKVVD VEEQQLE	36.177	0	0.099	SUMO Interaction
1653 - 1657	VNKRMSM VVSGL TPEEFML	6.098	0	0.817	SUMO Interaction
1676 - 1680	ARKHHIT LTNLI TEETTHV	2.689	0	0.926	SUMO Interaction
1791 - 1795	VQLCGAS VVKEL SSFTLGT	5.009	0	0.885	SUMO Interaction
1807 - 1811	LGTGVHP IVVVQ PDAWTED	62.804	0	0.005	SUMO Interaction
1838 - 1842	PVVTREW VLDSV ALYQCQE	5.035	0	0.935	SUMO Interaction
1854 - 1858	CQELDTY LIPQI PHSHY**	21.407	0	0.334	SUMO Interaction

G

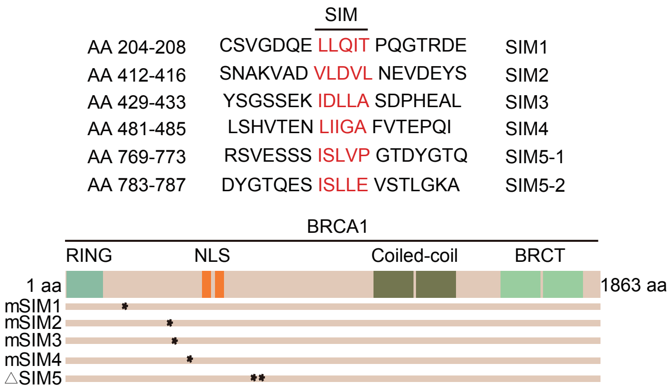

Fig. S5 PTEN chromatin loading is mediated by BRCA1 recruiting SUMOylated PTEN via its N-terminal SIM. (A) pT543-53BP1 were detected with immunoblot in U2OS cells in which BRCA1 was knocked down with siRNA or shRNA after treatment with Zeocin (400 μg/mL) or Etoposide (30 μM) for 1 h and recovery for indicated time. (B) Chromatin loaded PTEN was detected with immunoblot which was separated in BRCA1 knockdown DU145 cells with siBRCA1-1 after treatment with CPT (20 μM) for 1 h and recovery for indicated time. (C) Co-IP was performed to detected endogenous interaction between PTEN and BRCA1 in DU145 cell after CPT treatment.(D) Co-IP was performed to detect interaction between PTEN and BRCA1 in $293T^{senp-/-}$ cells which were overexpressed indicated plasmids for 48 h and treated with CPT (20 μM). (E-F) SIM location in BRCA1 were predicted with JASSA and GPS-SUMO software. We mainly focused on those SIMs marked with red font which were located in exon11 of BRCA1. (G) Schematic representation of mutation of selected SIMs. All those amino acids of predicted SIMs were mutated into alanine, except SIM5 (5-1 and 5-2) which had been deleted. Relative location of predicted SIMs in BRCA1 protein were shown at lower panel.
