# Supplementary material for: PTEN‐mediated dephosphorylation of 53BP1 confers cellular resistance to DNA damage in cancer cells
Source: Mol Oncol. 2023 Dec 12;18(3):580–605. doi: 10.1002/1878-0261.13563 (PMC10920079; doi:10.1002/1878-0261.13563)
Supplement: Supplementary file 6 — Fig. S6. Homologous recombination repair is impaired by SUMO‐deficient PTEN in vivo. [file MOL2-18-580-s008.pdf]

He_Fig. S6

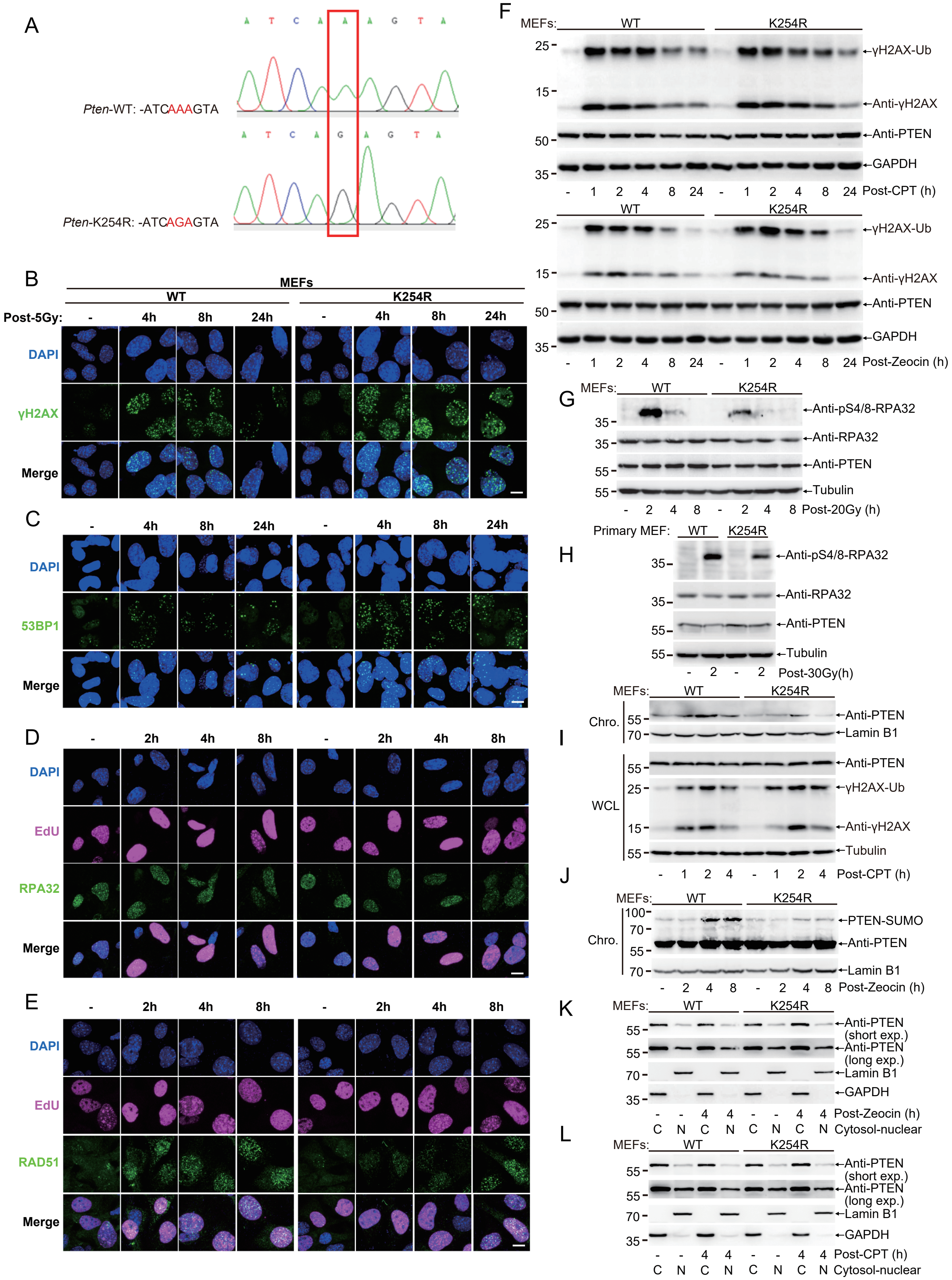
A
Pten-WT: -ATCAAAGTA
Pten-K254R: -ATCAGAGTA
A T C A A A G T A
A T C A G A G T A
B
MEFs
WT
K254R
Post-5Gy:
-
4h
8h
24h
DAPI
γH2AX
Merge
C
53BP1
D
2h
EdU
RPA32
E
RAD51
F
MEFs:
25
15
50
35
←γH2AX-Ub
←Anti-γH2AX
←Anti-PTEN
←GAPDH
- 1 2 4 8 24 - 1 2 4 8 24 Post-CPT (h)
- 1 2 4 8 24 - 1 2 4 8 24 Post-Zeocin (h)
G
←Anti-pS4/8-RPA32
←Anti-RPA32
←Tubulin
- 2 4 8 - 2 4 8 Post-20Gy (h)
Primary MEF:
H
- 2 - 2 Post-30Gy(h)
I
Chro.
WCL
←Lamin B1
- 1 2 4 - 1 2 4 Post-CPT (h)
J
←PTEN-SUMO
100
70
55
- 2 4 8 - 2 4 8 Post-Zeocin (h)
K
←Anti-PTEN (short exp.)
←Anti-PTEN (long exp.)
- - 4 4 - - 4 4 Post-Zeocin (h)
C N C N C N C N Cytosol-nuclear
L
- - 4 4 - - 4 4 Post-CPT (h)
C N C N C N C N Cytosol-nuclear

M

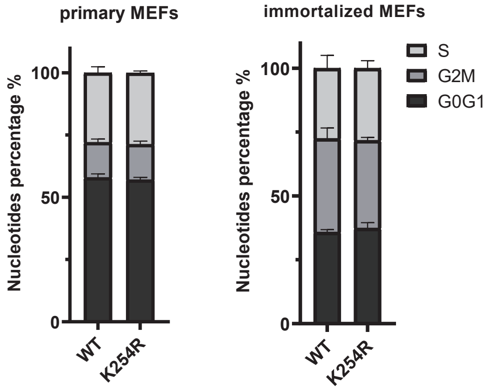

Fig. S6 HR repair is impaired by SUMO-deficient PTEN in vivo. (A) Sequencing of $Pten^{WT}$ and $PtenK^{254R}$ knockin mouse. (B-E) Representative images of 53BP1, γH2AX, RAD51 and RPA32 foci of MEFs corresponding to Fig. 6A-D which were treated with 5 Gy and recovery for indicated time. (F) Immunoblot of γH2AX from MEFs treated with CPT (20 μM) or Zeocin (200 μg/mL) for 1 h and recovery for indicated time. (G) Immunoblot of pS4/8-RPA32 from MEFs treated with 20 Gy and recovery for indicated time. (H) Immunoblot of pS4/8-RPA32 from primary MEFs treated with 30 Gy and recovery for indicated time.(I) Immunoblot of chromatin loaded PTEN which was separated from MEFs treated with CPT (20 μM) for 1 h and recovery for indicated time. (J) Immunoblot of chromatin loaded PTEN which was separated from MEFs treated with Zeocin (400 μg/mL) for 1 h and recovery for indicated time under harsh condition. (K, L) Nuclear-cytosol separation of MEFs post Zeocin (200 μg/mL) and CPT(20 μM) for 1 h and recovery for 4 h and then PTEN was detected with immunoblot. (M) Cell cycle profile was detected in primary and immortalized MEFs.
