# Supplementary material for: PTEN‐mediated dephosphorylation of 53BP1 confers cellular resistance to DNA damage in cancer cells
Source: Mol Oncol. 2023 Dec 12;18(3):580–605. doi: 10.1002/1878-0261.13563 (PMC10920079; doi:10.1002/1878-0261.13563)
Supplement: Supplementary file 7 — Fig. S7. Blocking PTEN SUMOylation pathway sensitizes tumor cells to DNA damage reagents. [file MOL2-18-580-s006.pdf]

He_Fig. S7

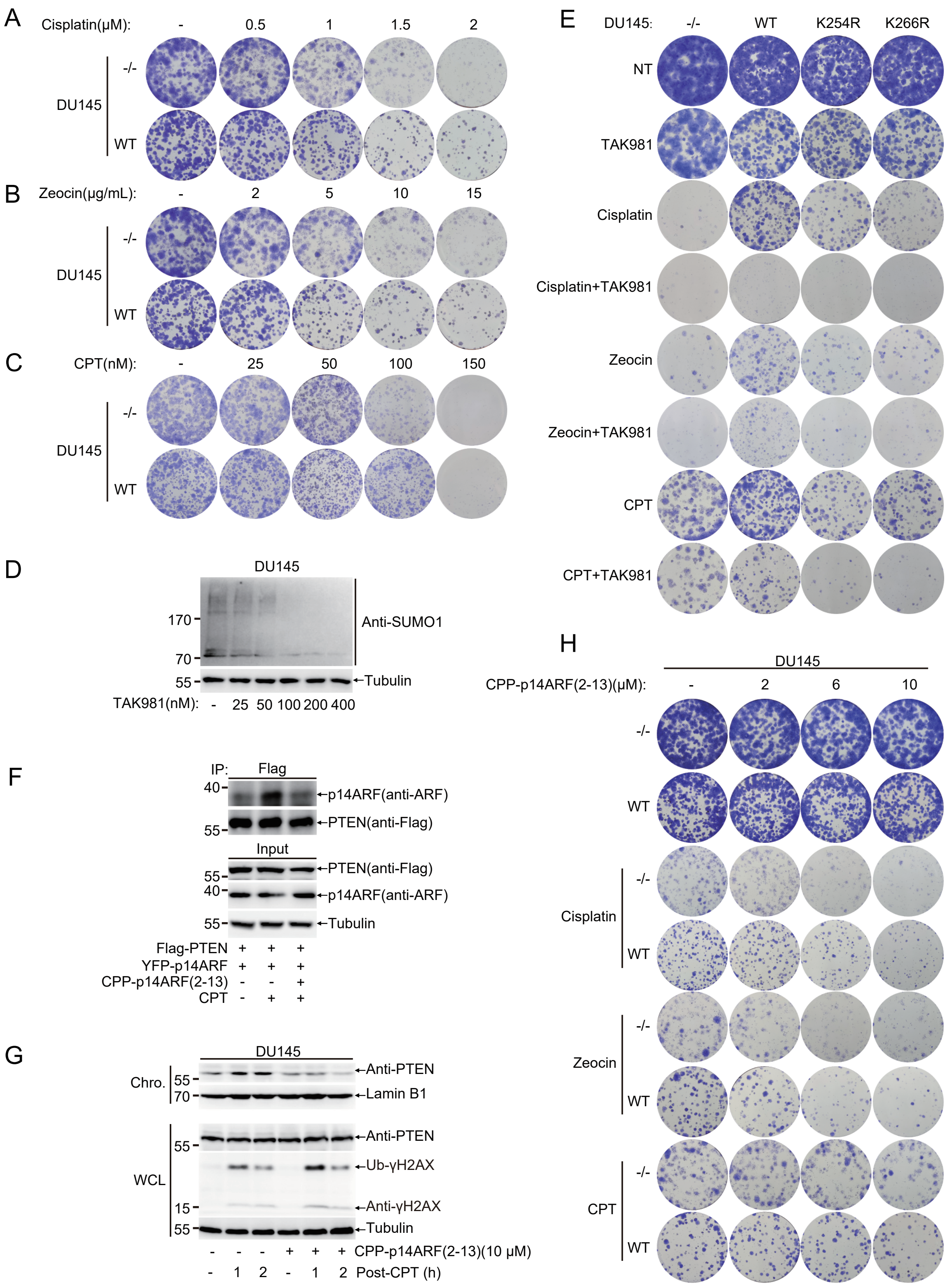
A
Cisplatin(μM): - 0.5 1 1.5 2
DU145
-/-
WT
B
Zeocin(μg/mL): - 2 5 10 15
DU145
-/-
WT
C
CPT(nM): - 25 50 100 150
DU145
-/-
WT
D
DU145
170
70
55
Anti-SUMO1
Tubulin
TAK981(nM): - 25 50 100 200 400
E
DU145: -/- WT K254R K266R
NT
TAK981
Cisplatin
Cisplatin+TAK981
Zeocin
Zeocin+TAK981
CPT
CPT+TAK981
F
IP: Flag
40
55
p14ARF(anti-ARF)
PTEN(anti-Flag)
Input
55
40
55
PTEN(anti-Flag)
p14ARF(anti-ARF)
Tubulin
Flag-PTEN + + +
YFP-p14ARF + + +
CPP-p14ARF(2-13) - - +
CPT - + +
G
DU145
Chro.
55
70
Anti-PTEN
Lamin B1
WCL
55
15
55
Anti-PTEN
Ub-γH2AX
Anti-γH2AX
Tubulin
- - - + + + CPP-p14ARF(2-13)(10 μM)
- 1 2 - 1 2 Post-CPT (h)
H
DU145
CPP-p14ARF(2-13)(μM): - 2 6 10
-/-
WT
Cisplatin
-/-
WT
Zeocin
-/-
WT
CPT
-/-
WT

Fig. S7 Blocking PTEN SUMOylation pathway sensitizes tumor cells to DNA damage reagents. (A-C) Representative colony images of DU145-PTEN$^{-/-}$ and PTENWT cells treated with or without different doses of Cisplatin, Zeocin or CPT corresponding to Fig. 7A-C. (D) Immunoblot of total SUMO1 conjugates in DU145 cells treated with different doses of TAK981. (E) Representative colony images corresponding to Fig. 7D-G. (F) 293T cells were transfected with Flag-PTEN and YFP-p14ARF for 48 h and then treated with or without CPP-p14ARF(2-13) for 4 h. CPT (20 μM) was then added into culture medium for 1 h. Cells were collected after another 2 h. Interaction between PTEN and p14ARF were identified with Co-IP. (G) DU145-PTEN-WT cells were treated with or without CPP-p14ARF(2-13) for 4 h and then cells were treated with CPT (20 μM) for 1 h and recovery indicated time. Chromatin loaded PTEN was separated and detected with immunoblot. (H) Representative colony images corresponding to Fig.7I-L. 500 cells were seeded in 12-well plate for all colony assays except (C) in which 1000 cells were seeded at the beginning.
